# Supplementary material for: Comparative multiomics analysis of cell physiological state after culture in a basket bioreactor
Source: Sci Rep. 2022 Nov 23;12:20161. doi: 10.1038/s41598-022-24687-4 (PMC9686226; doi:10.1038/s41598-022-24687-4)
Supplement: Supplementary file 1 — Supplementary Information 1. [file 41598_2022_24687_MOESM1_ESM.zip › raw data/Transcriptomic raw data/6.Enrichment/1.GO/TE1vsTC1/UP/TE1vsTC1.up_GObar_cc.pdf]

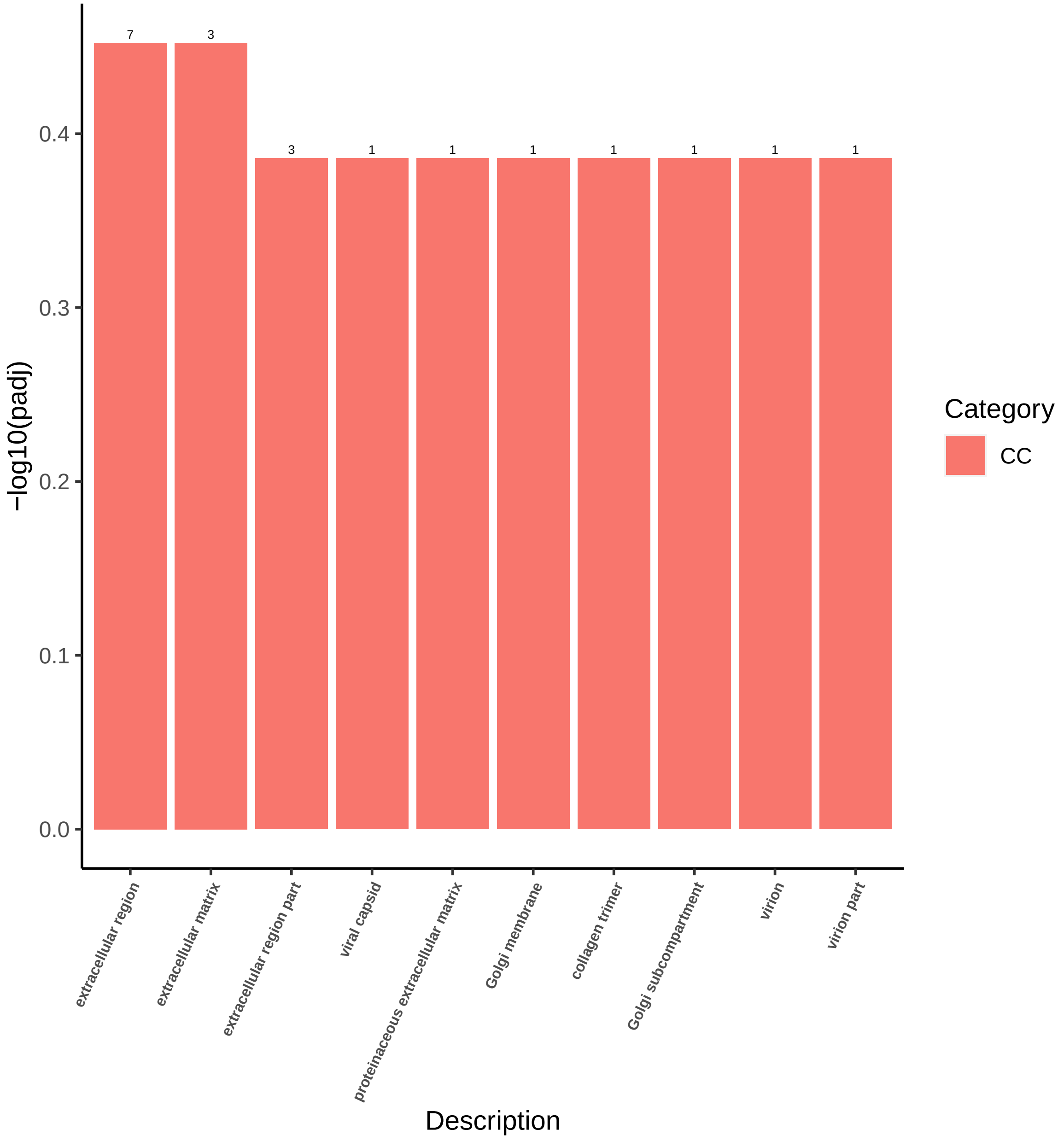

7
3
3
1
1
1
1
1
1
1
0.4
0.3
0.2
0.1
0.0
−log10(padj)
extracellular region
extracellular matrix
extracellular region part
viral capsid
proteinaceous extracellular matrix
Golgi membrane
collagen trimer
Golgi subcompartment
virion
virion part
Description
Category
CC
